# The effect of fecal bile acids on the incidence and risk-stratification of colorectal cancer: an updated systematic review and meta-analysis

**DOI:** 10.1038/s41598-024-84801-6

**Published:** 2025-01-04

**Authors:** Shaohui Yang, Yu Wang, Lijuan Sheng, Wei Cui, Chenyang Ma

**Affiliations:** 1 Department of Colorectal Surgery, Ningbo Medical Center Lihuili Hospital, Ningbo, 315000 China; 2 Gulou Street Community Health Service Center, Ningbo, 315000 China

**Keywords:** Colorectal cancer, Adenoma, Bile acid, Meta-analysis, Colorectal cancer, Tumour biomarkers

## Abstract

Recent studies suggest the role of gut microbes in bile acid metabolism in the development and progression of colorectal cancer. However, the surveys of the association between fecal bile acid concentrations and colorectal cancer (CRC) have been inconsistent. We searched online to identify relevant cross-sectional and case-control studies published online in the major English language databases (Medline, Embase, Web of Science, AMED, and CINAHL) up to January 1, 2024. We selected studies according to inclusion and exclusion criteria and extracted data from them. RevMan 5.3 was used to perform the meta-analyses. In CRC risk meta-analysis, the effect size of CA (cholic acid), CDCA (chenodeoxycholic acid), DCA (deoxycholic acid), and UDCA (ursodeoxycholic acid) were significantly higher (CA: standardized mean difference [SMD] = 0.41, 95% confidence interval [CI]: 0.5–0.76, *P* = 0.02; CDCA: SMD = 0.35, 95% CI: 0.09–0.62, *P* = 0.009; DCA: SMD = 0.33,95% CI: 0.03–0.64, *P* = 0.03; UDCA: SMD = 0.46, 95% CI: 0.14–0.78, *P* = 0.005), and the combined effect size was significantly higher in the high-risk than the low-risk CRC group (SMD = 0.36, 95% CI: 0.21–0.51, *P* < 0.00001). In the CRC incidence meta-analysis, the effect sizes of CA and CDCA were significantly higher (CA: SMD = 0.42, 95% CI: 0.04–0.80, *P* = 0.03; CDCA: SMD = 0.61, 95% CI: 0.26–0.96, *P* = 0.00079), and their combined effect size was also significantly higher in the high-risk compared to low-risk CRC group (SMD = 0.39, 95% CI: 0.09–0.68, *P* = 0.01). Only one cross-sectional study suggested a higher concentration of CDCA, DCA, and UDCA in the stool of the CRC high-risk group than the low-risk group. These findings indicate that higher fecal concentrations of bile acid may be associated with a higher risk/incidence of CRC.

**Supplementary Information:**

The online version contains supplementary material available at 10.1038/s41598-024-84801-6.

## Introduction

Colorectal cancer (CRC) is a common malignant tumor of the digestive system, with the third highest morbidity and mortality rate^1^. In CRC pathophysiology, colorectal adenomas are precursors in most cases of CRC. Adenoma is considered a risk factor for CRC^2^, and colorectal adenomas are subdivided into conventional adenomas and sessile serrated polyps^3^. A recent study showed that the gut microbiota might be essential in this progression during the adenoma-CRC transition through several signaling pathways and organismal influences^4^.

A possible correlation between fecal bile acid (BA) levels and CRC has been suggested; however, meta-analyses have failed to show a possible correlation^5^. The role of BAs metabolism in gut microorganisms in the development of CRC has been revealed through research on gut microorganisms^6^. The presence of 10^13^–10^14^ microbiota in the gut, with the genetic potential to carry out thousands of chemical reactions, dramatically expands the body’s metabolic capacity. BAs are one of the most essential gut microbiota metabolites^7^. BAs in the intestine solubilize dietary lipids and promote their excretion and absorption. They are also hormones that regulate BA biosynthesis, lipid and glucose homeostasis, and immune signaling^8^. A significant increase in DCA was found in the feces of patients with polypoid adenomas, and a microorganism (*Bilophila wadsworthia*) was significantly correlated with DCA levels^9^. It utilizes taurine-bound sulfite to reduce BA and promotes genetic susceptibility to colitis in interleukin 10^−/−^ mice^10^. The gut microbiota-BA axis plays an essential role in the occurrence and development of intestinal diseases^11^.

Several cross-sectional and case-control studies have attempted to find a relationship between fecal BAs and CRC; however, their conclusions have not been consistent. More conclusive assessment of fecal BA content between CRC patients and healthy people is needed, and systematic comparisons between countries and regions are lacking due to differences in dietary habits and fecal composition.

With the addition of recent studies, there is a strong need to perform an updated systematic analysis of fecal BA concentrations and CRC risk/incidence to understand their relationship. Therefore, all the studies included in this study were observational, and the central issue was the relationship between fecal BAs and the risk/incidence of CRC. In a survey of CRC risk, the data were derived from cross-sectional studies among specific populations and case-control studies on the presence or absence of risk factors (adenomatous polyps). Studies on the incidence of CRC are based on comparisons between CRC patients and healthy people (or non-CRC patients). The chemical compositions studied included a variety of joint BAs (cholic acid, chenodeoxycholic acid, deoxycholic acid, lithocholic acid, and ursodeoxycholic acid) and primary, secondary, and total BAs. It should be noted that the primary, secondary, and total BA data are directly obtained from various studies rather than calculated by addition.

## Methods

This systematic review and meta-analysis followed the Preferred Reporting Items for Systematic Reviews and Meta-Analysis reporting guidelines. The study is registered in the PROSPERO database (registration code: CRD42024533773).

### Literature search

An online search identified relevant cross-sectional and case-control studies published online in the major English language databases (Medline, Embase, Web of Science, AMED, and CINAHL). The papers search for this study included only English peer-reviewed articles published before January 1st, 2024.

Steps for searching: (I) Search for articles, systematic reviews, and meta-analyses from the databases, and (II) Analyze and summarize subject words and keywords. The search terms were: “Bile Acids,” “Bile Salts,” and “Colorectal Neoplasms,” “Colonic Neoplasms,” “colorectal cancer” or “colon cancer,” “colorectal carcinoma” or “colon carcinoma,” “colorectal neoplasm,” “colon neoplasm,” “colorectal neoplasia” or “colon neoplasia,” “Colorectal Carcinomas.” Details of the search strategy: ((Bile Acid) OR (Bile Salt)) AND ((Colorectal Neoplasms) OR (Colonic Neoplasms) OR (colorectal cancer) OR (colon cancer) OR (colorectal carcinoma) OR (colon carcinoma) OR (colorectal neoplasm) OR (colon neoplasm) OR (colorectal neoplasia) OR (colon neoplasia) OR (Colorectal Carcinomas)) NOT ( (rats) OR (mouse)) OR (mice) OR (murine)) AND (humans [Filter]) (III) Further search for references and associated papers. (IV) Screening of articles according to the inclusion and exclusion criteria.

### Inclusion and exclusion criteria

Cross-sectional and case-control studies of fecal BA levels in patients with CRC or those at high risk for CRC have been officially published as of 1 January 2024. The studies used similar objectives and methods to compare fecal BA levels in patients with CRC or those at high risk for CRC with healthy people. The case-control study consisted of patients with CRC confirmed by endoscopic pathology as a case group and patients without CRC as a control group. Information on their fecal BA content was collected and analyzed. In the cross-sectional study, patients at high risk for CRC identified through epidemiological investigation served as the case group.

In contrast, a comparable group of non-high-risk CRC patients within the same time and scope was risk colorectal cancer patients within the same time and scope were selected as the control group. The primary outcome of the studies was to investigate the contents and types of various BAs in the feces of the case or control groups. The local ethics committee approved all studies, and informed consent was obtained.

All studies were required to have original paper and extractable data available. For duplicate publications, smaller datasets (the total number of subjects is less than ten), studies with incomplete or contradictory information, and significant errors in statistical methods were excluded. Reviews, letters, case reports, animal studies, and studies with non-primary data were also excluded.

### Literature screening and data extraction

We imported all search records (including author names, dates, journal titles, and abstracts) into EndNote X7 and deleted duplicate records. Search results were screened according to “inclusion criteria” and “exclusion criteria,” and then basic experiments and animal experiments were excluded. Two researchers (Shaohui Yang and Yu Wang) did the study screening independently, and a third researcher (Wei Cui) would decide if there was disagreement. Finally, researchers (Lijuan Sheng and Chenyang Ma) read the study. They extracted the primary study information (e.g., author, year, region), study object information (e.g., number, age, gender), and detection indicators (e.g., detection method, fecal BA type, content).

### Literature quality evaluation

Two researchers evaluated study quality independently, and a third researcher would make the final decision if there were disagreements. The Newcastle-Ottawa Scale (NOS) was used for case-control and cross-sectional studies to evaluate study quality^12^. The difference was that cross-sectional studies used modified NOS for evaluation^13^. NOS scores range from 0 to 9; studies with 0–3, 4–6, and 7–9 were considered low, moderate, and high-quality, respectively. Data from studies with a high risk of bias were unreliable, so they were excluded.

### Statistical methods

The meta-analysis was performed using the RevMan 5.3 statistical software provided by www.cochrane.org. Various BA components were analyzed by subgroup analysis, and then the results of each subgroup analysis were combined for analysis. Because of the possible heterogeneity, a random effects model was applied. A fixed-effect model was used to analyze data with no significant heterogeneity (I^2^ < 50%). The data in each study were recorded differently and converted to “mean ± standard deviation” form before the analysis began. The expression of concentrations varied across studies; therefore, SMD was used for analysis. A funnel plot was drawn to detect publication bias. When *p* < 0.05, the difference was statistically significant.

One article^14^ had no calculable data (mean, standard deviation, standard error, or 95% CI) and only provided a statistical graph. We use online analytical tools (https://automeris.io/WebPlotDigitizer/) to extract the data from the statistical figures. Some articles had no specific data or statistical graphs, so they are considered qualitative analyses.

## Results

### Literature search results

#### Study selection

**Fig. 1:**
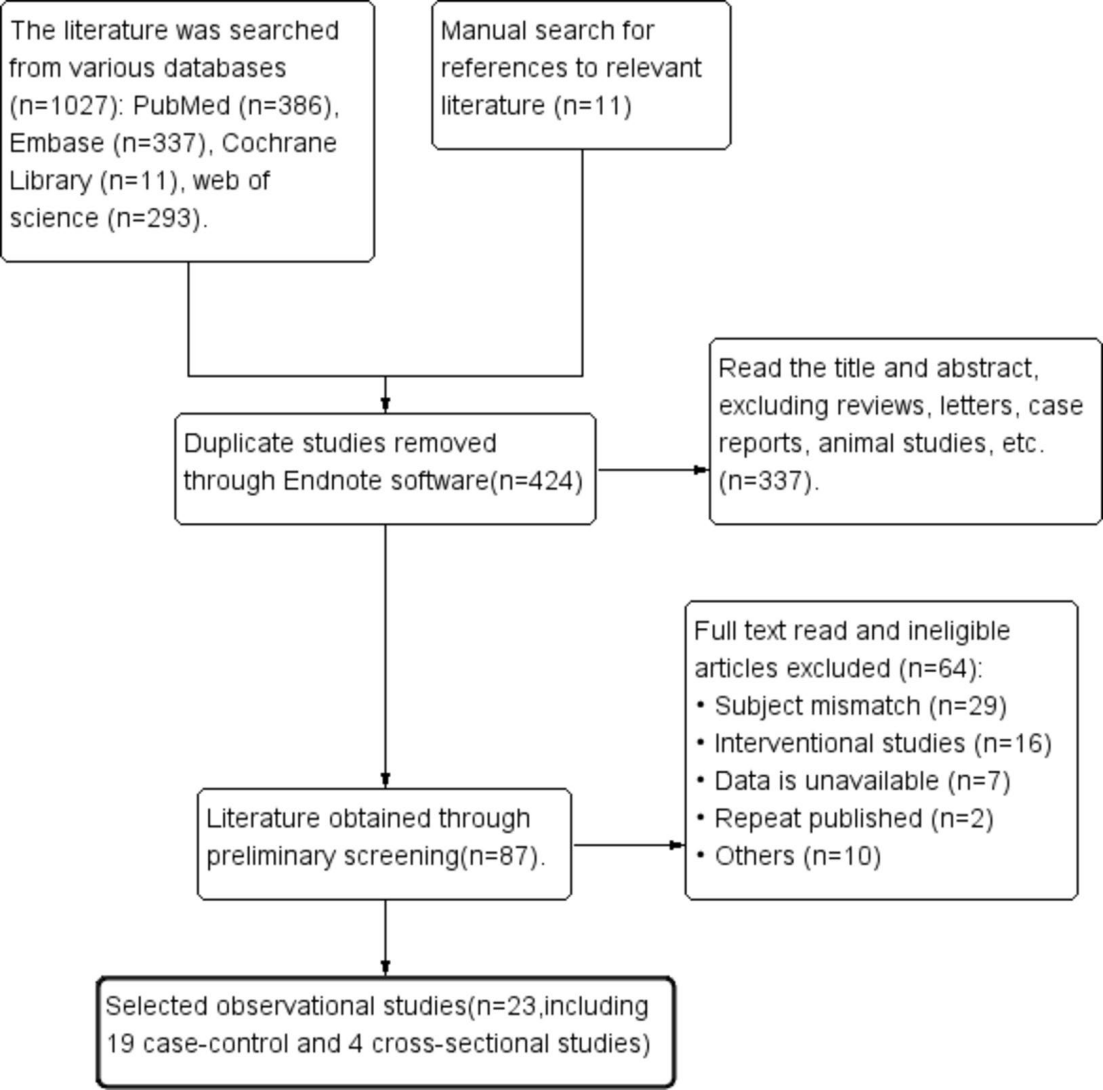
Flow chart of study search.

We searched 1027 relevant studies from various databases, and 11 were obtained from the manual detection of references of relevant studies. We imported all the data into EndNote, deleted the duplicate information, and received 424 studies. After reading the title and abstract of the study, we excluded reviews, letters, case reports, and animal studies. After reading the entire research and excluding unqualified studies, 23 were obtained, including 19 case-control^15–33^ and four cross-sectional studies^14,34–36^. The process of the study search is shown in Fig. 1.

### Quality evaluation of the study

Different NOS were used to evaluate the quality of 19 case-control^15–33^ and four cross-sectional^14,34–36^ studies. According to the scoring criteria, the quality evaluation results of 19 case-control studies^15–33^ were moderate and high. The quality evaluation results of four cross-sectional studies [14, 34–36] were all high-quality, showing that their data results are reliable (Tables 1 and 2).

**Table 1:** Details of the results of the quality evaluation of the case-control study.

Study	Selection				Comparability	Exposure			Total scores
	Adequate definition of cases	Representativeness of the cases	Selection of controls	Definition of controls	Control for important factors or additional factor	Ascertainment of exposure	The same method of ascertainment for cases and controls	Non-Response rate	
Boutron-Ruault et al. (2005)	★	★	★	★	★★	★	★	★	9
Korpela et al. (1988)	★			★	★	★	★	★	6
Chen et al. (2021)		★	★	★	★	★	★	★	7
TORII et al. (2019)	★		★	★	★	★	★	★	7
Reddy et al. (1977)	★			★	★	★	★	★	6
Owen et al. (1986)	★			★	★	★	★	★	6
Kaibara et al. (1983)	★			★	★	★	★	★	6
Owen et al. (1987)	★	★		★	★	★	★	★	7
Meance et al. (2003)	★	★	★	★	★	★	★	★	8
Owen et al. (1992)	★			★	★	★	★	★	6
Hill et al. (1987)	★			★	★	★	★	★	6
Breuer et al. (1986)	★			★	★	★	★	★	6
Breuer et al. (1985)	★			★	★	★	★	★	6
Murray et al. (1980)	★			★	★	★	★	★	6
Weir et al. (2013)	★	★	★	★	★	★	★	★	8
Hill et al. (1975)	★			★	★	★	★	★	6
Hikasa et al. (1984)	★			★	★	★	★	★	6
Tanida et al. (1984)	★			★	★	★	★	★	6
Kok et al. (1999)	★	★	★	★	★	★	★	★	8

**Table 2:** Details of the results of the quality evaluation of the cross-sectional study.

Study	Selection				Comparability	Outcome		Total scores
	Representativeness of the sample	Sample size	Response rate	Ascertainment of the exposure	Control for important factors or additional factor	Assessment of the outcome	Statistical test	
Ocvirk et al. (2020)	★	★	★	★★	★	★	★	8
Ou et al. (2013)	★	★	★	★★	★	★	★	8
Ou et al. (2012)	★	★	★	★★		★	★	7
Katsidzira et al. (2019)	★	★	★	★★	★★	★	★	9

### Characteristics of the selected study

The studies were divided into quantitative and qualitative studies according to the data availability for calculation (Table 3). Among the quantitative studies, three were cross-sectional studies^14,34,36^ (Table 3), and the remainder were case-control studies. One study was a cross-sectional study^35^ (Table 3), and five were case-control studies^15,19,20,28,31^, among six qualitative studies^15,19,20,28,31,35^. Cross-sectional studies compared people at high and low risk of CRC, so we identified all cross-sectional studies as CRC risk categories. In the case-control studies, seven^20,22,27–30,33^ were CRC incidence category due to the comparison of CRC cases and healthy controls, 11 studies^15,17–19,21,23–26,31,32^ were considered a CRC risk category because of “adenomatous polyps patients vs. healthy controls,” and one study comparing CRC cases and adenomatous polyps patients with healthy controls were considered as CRC incidence/risk categories^16^. These studies were conducted in multiple regions and countries (including China, the United Kingdom, the United States, France, Japan, Germany, and Zimbabwe) and included 1,265 patients and healthy people. All subjects had explicit inclusion and exclusion criteria. Endoscopy and pathology identified and diagnosed CRC/adenomatous polyp cases and healthy controls. In all case-control studies, the BA conditions of the case and control groups were described to ensure they were comparable. In cross-sectional studies, two similar populations were compared at a specific time and within a particular area to find the cause.

**Table 3:** Study characteristics.

Data	Study	Country	Study population (number of subjects)^a^	Age (years)^b^	Sex female/male	Measurement techniques^c^	Measured BAs^d^	Analysis category^e^
Quantitative	Boutron-Ruault et al. (2005) ^30^	French	Adenoma (50, large (18) and small (32)) vs. HC (44) ^f^	57.35 ± 8.41 vs. 52.5 ± 8.77^g^	(15/35, 4/14 and 11/21 ) vs. 23/21	GC-MS	CA, CDCA, DCA, LCA, UDCA, PBAs, SBAs	Risk
	Korpela et al. (1988) ^26^	Finnish	CRC (9) vs. vegetarian (10) vs. omnivorous (10)	63.4 ± 4.05 vs. 57.5 ± 2.48 vs. 57.4 ± 1.53	9/0 vs. 10/0 vs. 10/0	GLC	Total	Incidence
	Chen et al. (2021) ^33^	China	AP (30) vs. HC (30)	53.23 ± 10.14 vs. 50.33 ± 10.87	10/20 vs. 17/13	Ion chromatography and UPLC-MS	CA, CDCA, DCA, LCA,	Risk
	Torll et al. (2019) ^32^	Japan	CRC (15) vs. HC (38)	–	7/8 vs. 21/17	HPLC-FL	CA, CDCA, DCA, LCA, UDCA	Incidence
	Reddy et al. (1977) ^16^	USA/Japan	CRC (31) vs. AP (13) vs. Other digestive diseases (9) American controls (34) vs. Japanese controls (12)	Average age: 58 vs. 38 vs. 49 vs. 46 (American and Japanese controls combined)	16/15 vs. 5/8 vs. 4/5 vs. 24/22 (American and Japanese controls combined)	GC	CA, CDCA, DCA, LCA, Total	Incidence/risk
	Owen et al. (1986) ^23^	UK	CRC (34) vs. Breast cancer (16) vs. HC (36)	–	–	GC	LCA, DCA, Total	Incidence
	Kaibara et al. (1983) ^18^	Japan	colon cancer (10) vs. rectal cancer (5) vs. HC (10)	59 ± 7 vs. 61 ± 5 vs. 58 ± 8	4/6 vs. 2/3 vs. 5/5	GC-MS	CDCA, DCA, LCA, PBAs, SBAs, Total	Incidence
	Owen et al. (1987) ^25^	UK	CRC (17) vs. HC (20)	63 ± 2 vs. 59 ± 2	5/12 vs. 10/10	GLC-MS	DCA, LCA, Total	Incidence
	Meance et al. (2003) ^29^	French	CRA (19) vs. HC (20)	55.0 (44–62) vs. 53.1 (26–70)	10/9 vs. 10/10	HPLC-MS	CA, CDCA, DCA, LCA, UDCA, PBAs, SBAs	Risk
	Owen et al. (1992) ^27^	UK	Polyps (68) vs. HC (24)	70 ± 1 vs. 63 ± 2	26/42 ± 13/11	GLC-MS	LCA, DCA, Total	Risk
	Hill et al. (1987) ^24^	UK	UC patients with carcinoma or definite dysplasia (14) vs. UC patients without dysplasia or carcinoma (88)	–	–	Modified enzyme assay method	Total	Incidence
	Breuer et al. (1986) ^22^	Germany	AP (12) vs. HC (12)	56.9 ± 2.1 vs. 55.2 ± 2.0	2/10 vs. 2/10	GLC	CA, CDCA, DCA, LCA, UDCA, PBAs, SBAs	Risk
	Breuer et al. (1985) ^21^	Germany	CRC (23) vs. HC (21)	62vs49	15/8 vs. 6/15	GLC	PBAs, SBAs, Total	Incidence
	Murray et al. (1980) ^17^	UK	CRC (37) vs. HC (36)	66 vs. 65	16/21 vs. 16/20	Hydroxysteroid dehydrogenase enzyme assay method	Total	Incidence
	**Ocvirk et al. (2020)** ^36^	**USA/African**	**Alaska Native (32) vs. rural African (21)**	**51.0 ± 8.9 vs. 53.3 ± 11.5**	**24/8 vs. 12/9**	**HPLC-MS**	**DCA**	**Risk**
	**Ou et al. (2013)** ^14^	**USA/African**	**African Americans (12) vs. native Africans (12)**	**58 ± 2.5 vs. 57 ± 1.9**	**9/3 vs. 8/4**	**LC-MS**	**CA**,** CDCA**,** DCA**,** LCA**	**Risk**
	**Ou et al. (2012)** ^34^	**USA/African**	**African Americans (12) vs. Caucasian Americans (10) vs. native Africans (13)**	**50–60**	–	**LC-MS**	**CA**,** CDCA**,** DCA**,** LCA**,** UDCA GCA**,** TCA**,** GCDCA**,** TCDCA**,** GDCA**,** TDCA**,	**Risk**
Qualitative	Weir et al. (2013) ^31^	USA	CRC (10) vs. HC (11)	63.7 ± 17.7 vs. 40.7 ± 14.6	2/8 vs. 8/3	GC-MS	UDCA	Incidence
	Hill et al. (1975) ^15^	UK	CRC (44) vs. patients with other diseases (90)	62.1vs. 53.6	20/24 vs. 39/51	–	Total	Incidence
	Hikasa et al. (1984) ^19^	Japan	CRC (14) vs. HC (14)	63.4 ± 8.5 vs. 63.5 ± 11.8	9/5 vs. 9/5	GLC-MS	Total	Incidence
	Tanida et al. (1984) ^20^	Japan	AP (13) vs. HC (13)	58 ± 14 vs. 54 ± 12	3/10 vs. 2/11	GC-MS	CA, CACD, DCA, LCA	Risk
	Kok et al. (1999) ^28^	Netherlands	AP-high risk (20) vs. AP-medium risk (19) vs. HC (25)	–	–	GC	CA, CACD, DCA, LCA, UDCA, PBAs, SBAs	Risk
	**Katsidzira et al. (2019)** ^35^	**Zimbabwe**	**Urban Zimbabweans (10) vs. Rural Zimbabweans (10)**	**65.3 ± 10.0 vs. 61.6 ± 8.1**	**5/5 vs. 5/5**	**HPLC-MS**	**CA**,** CACD**,** DCA**,** LCA**,** UDCA**	**Risk**

Cross-sectional studies are highlighted in bold, and case-control studies are not highlighted.

^a^AP, Adenomatous polyps HC: Healthy Control CRA: Colorectal adenomas.

^b^Presented as Means ± SD/Mean/Mean (min-max)/ (min-max).

^c^GC, gas chromatography; MS, mass spectrometry; GLC, gas-liquid chromatography; HPLC, high-performance liquid chromatography; UPLC, ultra-performance liquid chromatography; FL, fluorescence.

^d^CA, Cholic acid; CDCA, Chenodeoxycholic acid; DCA, Deoxycholic acid; LCA, Lithocholic acid; UDCA, Ursodeoxycholic acid; GCA, glycocholic acid; TCA, taurocholic acid; GCDCA, glycochenodeoxycholic acid; TCDCA, taurochenodeoxycholic acid; GDCA, glycodeoxycholic acid; TDCA, taurodeocycholic acid; PBAs: Primary bile acids; SBAs: Secondary bile acids; Total: Total bile acids.

^e^Refer to the text for the definition of risk and incidence category^12^.

^f^BAs were measured in a subset of these subjects [large/small adenoma (42) and HC (28)].

^g^Combined values of males and females.

Due to different eras, all studies used different detection methods and technologies, including gas chromatography, mass spectrometry, and gas-liquid chromatography. Therefore, the accuracy of various detection techniques was different, but the meta-analysis results were still valuable. The chemical components tested in each study were cholic acid, chenodeoxycholic acid, deoxycholic acid, lithocholic acid, and ursodeoxycholic acid. Primary bile, secondary bile, and total BAs were also included, but they were analyzed separately (Table 3).

### Data analyses

**Fig. 2:**
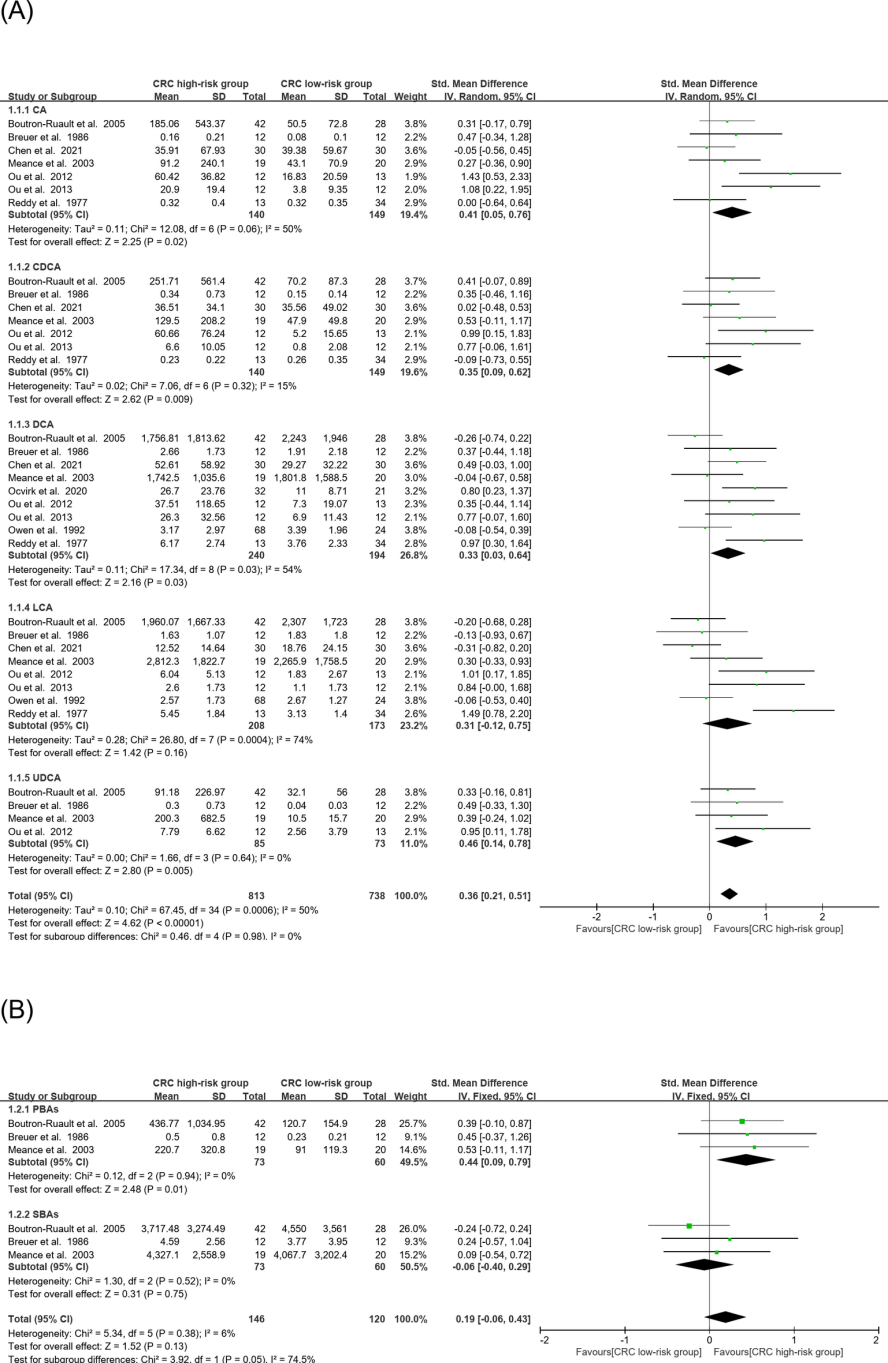

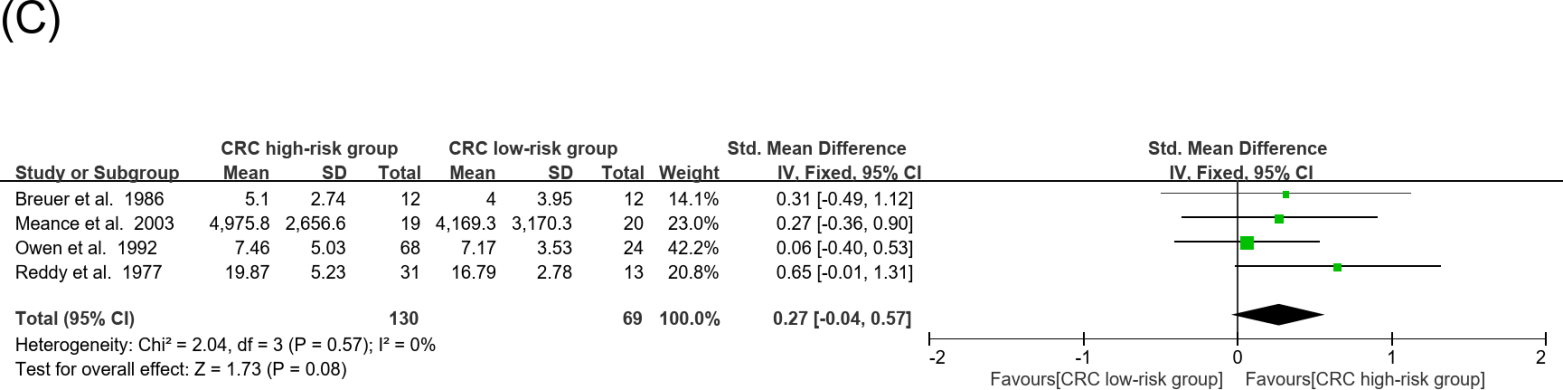
(**A**) Forest plots of a meta-analysis of the fecal concentrations of CA, CDCA, DCA, LCA, and UDCA in the CRC risk category. (**B**) Forest plots of a meta-analysis of the fecal concentrations of primary and secondary BAs in the CRC risk category. (**C**) Forest plots of a meta-analysis of the fecal concentrations of total BAs in the CRC risk category.

**Fig. 3:**
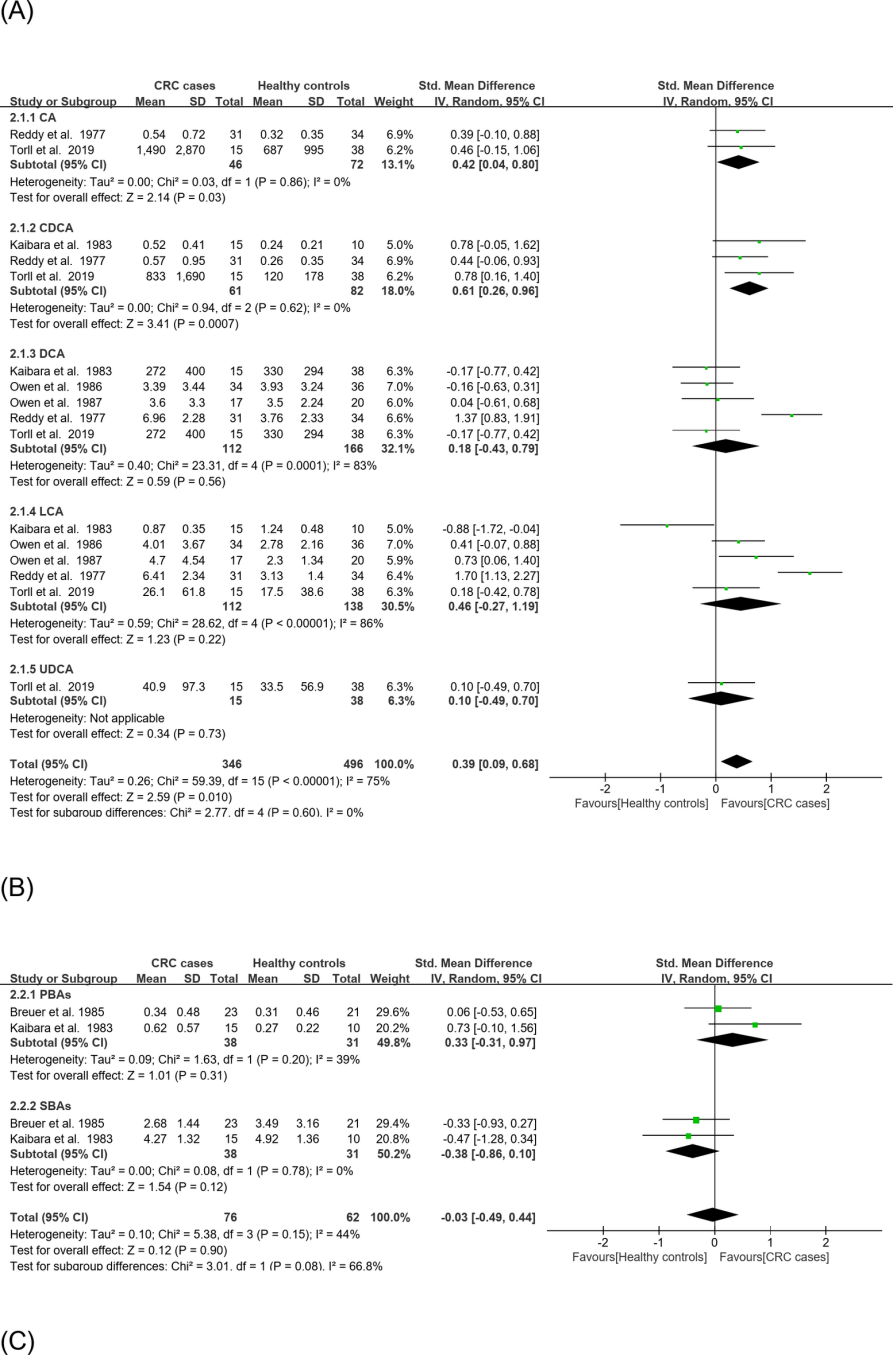

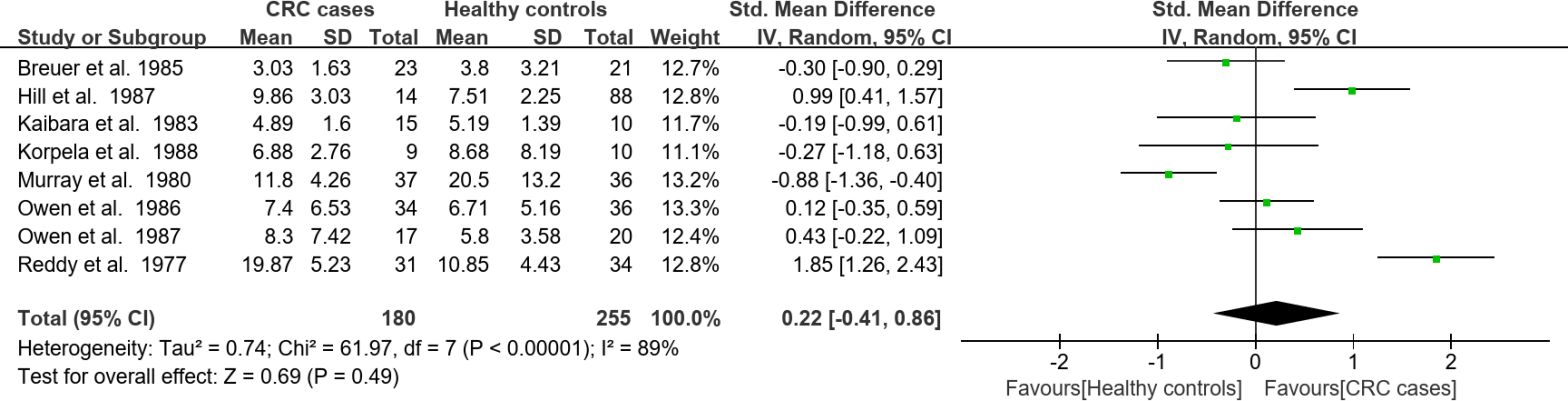
(**A**) Forest plots of a meta-analysis of the fecal concentrations of CA, CDCA, DCA, LCA, and UDCA in the CRC incidence category. (**B**) Forest plots of a meta-analysis of the fecal concentrations of primary and secondary BAs in the CRC incidence category. (**C**) Forest plots of a meta-analysis of the fecal concentrations of total BAs in the CRC incidence category.

The meta-analysis of the quantitative data extracted from the studies was presented in Figs. 2A–C and 3A–C. In the CRC risk meta-analysis, the effect size of CA, CDCA, DCA, and UDCA were significantly higher (CA: SMD = 0.41, 95% CI: 0.5–0.76, *P* = 0.02; CDCA: SMD = 0.35, 95% CI: 0.09–0.62, *P* = 0.009; DCA: SMD = 0.33, 95% CI: 0.03–0.64, *P* = 0.03; UDCA: SMD = 0.46,95% CI: 0.14–0.78, *P* = 0.005), and the combined effect size was significantly higher in high-risk than the low-risk CRC group (SMD = 0.36, 95% CI: 0.21–0.51, *P* < 0.00001) (Fig. 2A). The effect size of primary BAs was significantly higher (SMD = 0.44, 95% CI: 0.09–0.71 *P* = 0.01); however, the combined effect size of primary and secondary BAs was not significantly higher (Fig. 2B). The effect size of total BAs was not significantly higher in the high-risk vs. the low-risk group (Fig. 2C). Total BA means collecting all the BA molecules, not only CA, CDCA, DCA, LCA, and UDCA, as were primary and secondary BAs.

In CRC incidence meta-analysis, the effect size of CA and CDCA were significantly higher (CA: SMD = 0.42, 95% CI: 0.04–0.80, *P* = 0.03; CDCA: SMD = 0.61,95% CI: 0.26–0.96, *P* = 0.00079), and their combined effect size was also significantly higher in high-risk compared to low-risk CRC group (SMD = 0.39, 95% CI: 0.09–0.68, *P* = 0.01) (Fig. 3A). The effect size of primary, secondary, and total BAs (including the combined effect size of primary and secondary BAs) all were not significantly higher in the high- vs. low-risk group (Fig. 3B, C).

Table 4 presents all the results of the random-effect model meta-analyses. Data with low heterogeneity were more suitable for the fixed-effect model^37^; therefore, we performed another meta-analysis using a fixed-effect model for data with I^2^ less than 50%, and the results showed the same conclusion as the random-effect model (Table 5).

**Table 4:** Summary of the outcomes of meta-analysis by random-effect model.

	Measured BAs	Number of studies	Heterogeneity (I^2^%, *P*-value)	SMD [95% CI]	*P*-value
CRC risk	CA	7	50, 0.06	0.41 [0.05, 0.76]	0.02
	CDCA	7	15, 0.32	0.35 [0.09, 0.62]	0.009
	DCA	9	54, 0.03	0.33 [0.03, 0.64]	0.03
	LCA	8	74, 0.0004	0.31 [− 0.12, 0.75]	0.16
	UDCA	4	0, 0.64	0.46 [0.14, 0.78]	0.005
	Combined of BAs*	9	50, 0.0006	0.36 [0.21, 0.51]	< 0.00001
	PBAs	3	0, 0.94	0.44 [0.09, 0.79]	0.01
	SBAs	3	0, 0.52	− 0.06 [− 0.40, 0.29]	0.75
	Combined of PBAs and SBAs	3	6, 0.38	0.19 [− 0.06, 0.45]	0.14
	Total BAs	4	0, 0.57	0.27 [− 0.04, 0.57]	0.08
CRC incidence	CA	2	0,0.86	0.42 [0.04, 0.80]	0.03
	CDCA	3	0, 0.62	0.61 [0.26, 0.96]	0.0007
	DCA	5	83, 0.0001	0.18 [− 0.43, 0.79]	0.56
	LCA	5	86, < 0.00001	0.46 [− 0.27, 1.19]	0.22
	UDCA	1	–	0.10 [− 0.49, 0.70]	0.73
	Combined of BAs*	5	75, < 0.00001	0.39 [0.09, 0.68]	0.01
	PBAs	2	39, 0.20	0.33 [− 0.31, 0.97]	0.31
	SBAs	2	0, 0.78	− 0.38 [− 0.86, 0.10]	0.12
	Combined of PBAs and SBAs	2	44, 0.15	− 0.03 [− 0.49, 0.44]	0.90
	Total BAs	8	89, < 0.00001	0.22 [− 0.41, 0.86]	0.49

*CA* Cholic acid, *CDCA* Chenodeoxycholic acid, *DCA* Deoxycholic acid, *LCA* Lithocholic acid, *UDCA* Ursodeoxycholic acid, *PBAs* Primary bile acids, *SBAs* Secondary bile acids.

*BAs included CA, CDCA, DCA, LCA, and UDCA.

**Table 5:** Summary of the outcomes of the meta-analysis by fixed-effect model.

	Measured BAs	Number of studies	Heterogeneity (I^2^%, *P*-value)	SMD [95% CI]	*P*-value
CRC risk	CDCA	7	15, 0.32	0.34 [0.10, 0.58]	0.005
	UDCA	4	0, 0.64	0.46 [0.14, 0.78]	0.005
	PBAs	3	0, 0.94	0.44 [0.09, 0.79]	0.01
	SBAs	3	0, 0.52	− 0.06 [− 0.40, 0.29]	0.13
	Combined of PBAs and SBAs	3	6, 0.38	0.19 [− 0.06, 0.43]	0.38
	Total BAs	4	0, 0.57	0.27 [− 0.04, 0.57]	0.08
CRC incidence	CA	2	0, 0.86	0.42 [0.04, 0.80]	0.03
	CDCA	3	0, 0.62	0.61 [0.26, 0.96]	0.0007
	PBAs	2	39, 0.20	0.29 [− 0.20, 0.77]	0.24
	SBAs	2	0, 0.78	− 0.38 [− 0.86, 0.10]	0.12
	Combined of PBAs and SBAs	2	44, 0.15	− 0.05 [− 0.39, 0.29]	0.79

*CA* Cholic acid, *CDCA* Chenodeoxycholic acid, *DCA* Deoxycholic acid, *LCA* Lithocholic acid, *UDCA* Ursodeoxycholic acid, *PBAs* Primary bile acids, *SBAs* Secondary bile acids.

In qualitative studies, the results of only one cross-sectional study^35^ suggested that the concentration of CDCA, DCA, and UDCA in the stool of the CRC high-risk group than in the low-risk group, similar to Hill et al.^15^. However, Weir et al. suggested that the level of UDCA in the stool of HC was higher. In addition, the results of the other three cross-sectional and case-control studies did not show differences in fecal BA levels between CRC high-risk vs. low-risk groups or CRC vs. HC. Some of the qualitative analysis results were consistent with our meta-analysis.

### Publication bias detection

**Fig. 4:**
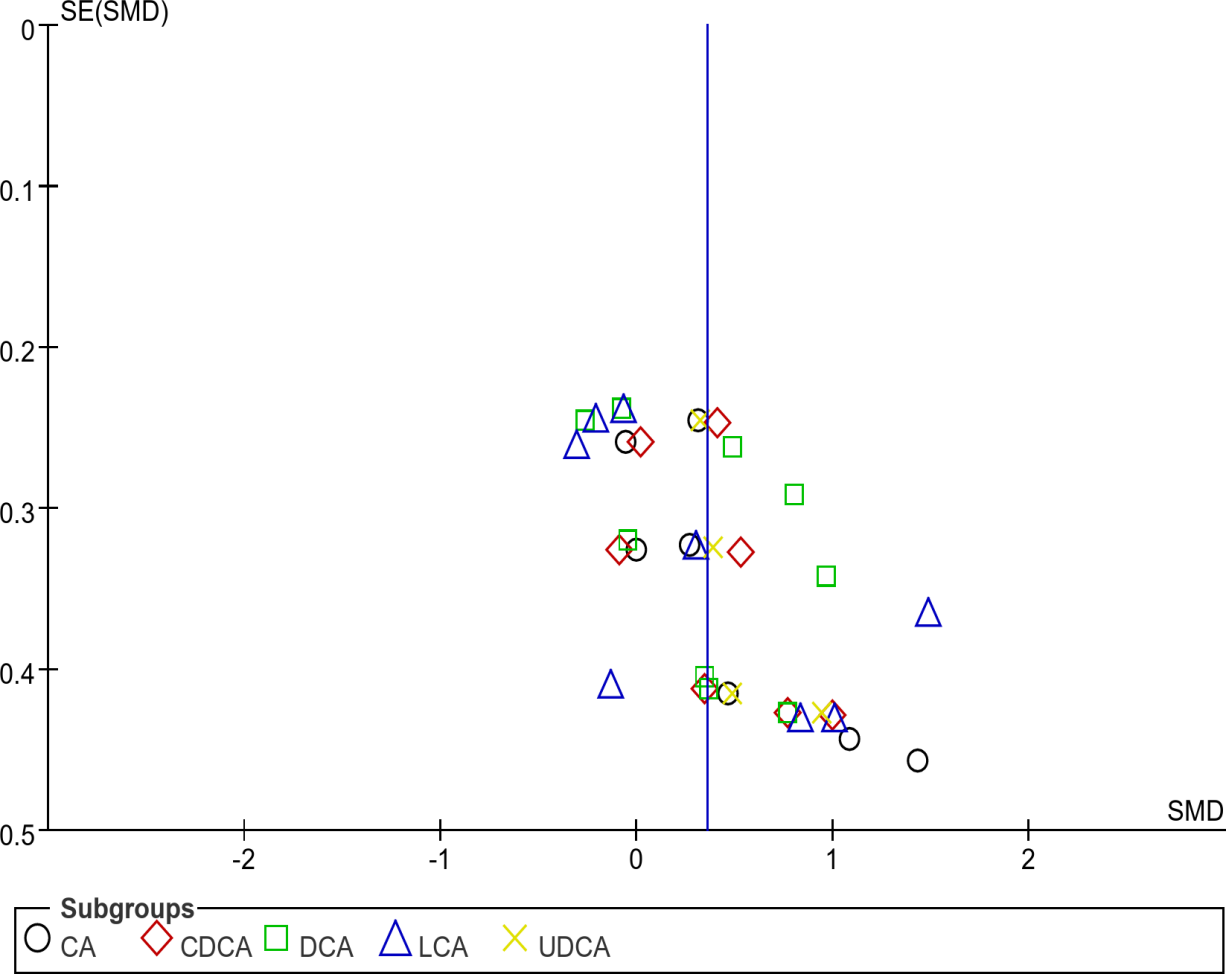
Funnel plot of the BAs in stool.

We used a funnel plot to detect publication bias for several primary BAs in stool, and the results are shown in Fig. 4. The asymmetric distribution of each point in the funnel plot suggested the presence of publication bias in this study.

## Discussion

Forty years ago, some scholars began studying the relationship between BA concentration in stool and CRC^16^. Since then, similar clinical study results have been published but are limited to the different study designs and detection methods; the results of each study were. In recent years, clinical metagenomics and metabolomics studies have revealed the association between microbial participation in BA metabolism and intestinal diseases, and the correlation between fecal BAs and CRC has been given more attention^38^.

This systematic review and meta-analysis included several clinical studies investigating the relationship between the concentration of BAs in stool and the risk and incidence of CRC. We found that individuals with a high risk of CRC had a higher concentration of fecal BAs than those with a low risk in the CRC risk category, suggesting a potential association between fecal BAs and CRC development, and this finding was confirmed in the CRC incidence category. Some types of BAs can be classified as primary or secondary BAs. In the specific analysis and calculation, the primary or secondary BA data was directly obtained from the original study rather than by adding the data of BAs. This finding could have occurred because different studies adopted different detection methods; some BA detection methods needed more accuracy in earlier studies, and superimposed calculations may have caused inaccurate data.

BAs are produced mainly by the liver and released into the intestine by the gallbladder, and it has long been found that cholecystectomy impacts the incidence of CRC^39^. Therefore, it is easy to think of BAs’ role in CRC development^40^. Subsequent studies have revealed the influence of BAs on intestinal microecology after cholecystectomy, changing the composition and function of intestinal microbiota and finally leading to the occurrence of CRC^41^. The specific mechanism may be that after gallbladder removal, the regulation of BA excretion is weakened, and the biophysical properties, fluid content, pH, and even immune status in the gut will change, thus providing favorable or harmful growth conditions for certain bacteria^42^. These studies provide evidence that BAs exert a more comprehensive range of biological activity than initially realized, and whether BA metabolism in the gut microbiome affects health or disease depends on the type and concentration of BAs^43^. For example, lithocholic acid, which is produced by chenodeoxycholic acid through intestinal microbial metabolism and dehydroxylation, is toxic to liver cells and has been linked to the development of colon cancer^44^. Several lines of evidence point to a robust association between the BA-microbiota crosstalk and CRC risk, development, and progression^45^. Therefore, it is necessary to study the relationship between them.

Several mechanistic studies have explained the relationship between fecal BA concentration and the risk and incidence of CRC^9,46,47^. In a meta-analysis of geographically and technically diverse fecal shotgun metagenomic studies of CRC, genes involved in secondary BA synthesis were found to be upregulated in CRC metagenomes, promoting secondary BA production, suggesting a metabolic link between CRC-associated gut microbes and the meat diet^46^. The highly active bile salt hydrolase in Bacteroides can promote the production of free deoxycholic acid and lithocholic acid. Further activation of the G-protein-coupled BA receptor, which increases β-catenin-regulated CCL28 expression in CRC, leads to intratumor immunosuppression via CD25FOXP3 T + + reg cells^48^. By studying gut microbiota and metabolites in stool, in addition to changes in several microorganisms (*Fusobacterium nucleatum*,* Atopobium parvulum*, and *Actinomyces odontolyticus*), multiple polypoid adenomas or intramucosal carcinomas BAs (including deoxycholate) were significantly elevated, suggesting changes in the microbiome and metabolome in the early stages of CRC^9^. Because fecal BAs can be absorbed into the bloodstream through the intestine, plasma levels of seven binding BA metabolites were positively associated with CRC risk before CRC diagnosis in a prospective case-control study (glycocholic acid, taurocholic acid, glycochenodeoxycholic acid, taurochenodeoxycholic acid, and glycohyocholic acid, glycodeoxycholic acid and taurodeoxycholic acid). No correlation was found between unconjugated and tertiary BAs^47^.

With the research on gut microbiota, the existence and influence of gut microbiota-BA axis in the body have been gradually revealed^49,50^. In addition to participating in the enterohepatic circulation, BA in the gut is also subject to microbiota-mediated biotransformation, which increases and changes the types and content of BA^51^. Gut microbiota mediates the biotransformation of BA is mediated by enzymatic catalysis, including deconjugation, 7α/β-dehydroxylation, oxidation/epimerization, esterification, desulfation, and conjugation^52^. BSH catalyzes the amide bond cleavage in BAs, releasing free BAs (such as CA and CDCA), along with glycine or taurine, which serve as nutrients for the gut microbiota^53^. BSH plays a crucial role in BA-mediated signaling by regulating lipid uptake, glucose metabolism, and energy homeostasis^53^.

Conversely, changes in the type and content of BA will also change the composition and abundance of the gut microbiota because BA is a crucial antibacterial substance in the gut, which can achieve antibacterial effects by destroying cell membranes^54^. LCA and its derivatives have been found to have antibacterial effects on *Escherichia coli*, *Staphylococcus aureus*, *Bacillus cereus*, and *Pseudomonas aeruginosa*^55^. Therefore, gut microbiota and BA have a subtle relationship of mutual dependence and restriction. Recent studies have revealed that the gut microbiota-BA axis can affect human physiological processes through various mechanisms and disease processes of the digestive, circulatory, and reproductive systems, a potential target for disease treatment^50,52,56^.

After including the latest clinical studies, our meta-analysis led to a different conclusion: most of the BAs (CA, CDCA, DCA, UDCA) in the feces of the CRC high-risk group were higher than those of the low-risk group. BAs (CA, CDCA) in CRC cases were also higher than those in healthy controls, and the difference in the combined effect size of the low-risk/high-risk group and CRC cases/healthy controls was statistically significant. However, the meta-analysis of primary, secondary, and total BAs had the same conclusion as previous studies, and no statistical difference was found between the two groups because no new studies were included^5^.

There were some limitations to this study. First, this meta-analysis included case-control and cross-sectional studies, likely to have unavoidable information bias and confounding bias^57^. Therefore, we designed two different NOS to evaluate the quality of the research and provide a more comprehensive understanding of the results of the meta-analysis. Secondly, the period of this meta-analysis is long. The detection methods used in each study are different, and the accuracy of the detection results is also different, which will inevitably affect the final analysis results. However, the number of studies included in this study must be more significant in order to perform subgroup analysis. In addition, these studies come from different countries and regions, and the subjects are different. Therefore, we must admit that is unavoidable heterogeneity is this study’s most significant limitation. Third, only English studies were included; thus, there is a risk of missing detection. Finally, the fecal composition of CRC patients at different stages can be analyzed and compared. In that case, meaningful data may be found, but no relevant study has been found.

The mechanism by which gut microorganisms influence disease through BA metabolism has also been revealed due to the continuous research on the role of gut microbiota in the development of colorectal tumors. Furthermore, the relationship between fecal BA level and CRC has been paid more attention^41^. The updated meta-analysis differs from previous results because it includes recent clinical studies. Our study showed that CRC and high-risk patients had higher concentrations of some BAs in their stool than healthy controls and low-risk patients. Because of the possible correlation between fecal BA concentration and colorectal risk/morbidity, more attention is likely to be paid to predicting and treating CRC.

## Electronic supplementary material

Below is the link to the electronic supplementary material.

Supplementary Material 1

## Data Availability

Some or all data are available from the corresponding author by request.
